# Rational diagnoses of diabetes: the comparison of 1,5-anhydroglucitol with other glycemic markers

**DOI:** 10.1186/s40064-015-1389-5

**Published:** 2015-10-09

**Authors:** Umit Yavuz Malkan, Gursel Gunes, Ahmet Corakci

**Affiliations:** Department of Internal Medicine, School of Medicine, Ufuk University, Ankara, Turkey; Department of Endocrinology, School of Medicine, Ufuk University, Ankara, Turkey

**Keywords:** 1,5-Anhydroglucitol, Glycated hemoglobin, Fructosamine, Prediabetes, Diabetes mellitus, Diagnosis

## Abstract

Diabetes mellitus (DM) is a frequently encountered disease with important morbidity and mortality. The aim of this study is to document the importance of 1,5-anhydroglucitol (1,5-AG) for the diagnosis of prediabetes and DM, as well as to compare the 1,5-AG with other glycemic markers in order to understand which one is the better diagnostic tool. Between April 2012 and December 2012, 128 participants enrolled in the study. Participants were split into five groups that are IFG, IGT, IFG+IGT, diabetic and control groups by their OGTT results. The diagnostic value of markers was compared by ROC (receiver operating characteristic) method. The mean serum 1,5-AG levels in the diabetic group (33.38 nmol/ml) were lower than, IFG (59.83 nmol/ml), IGT (54.44 nmol/ml), IFG+IGT (51.98 nmol/ml) and control groups (73.24 nmol/ml). When analyzed in the total study population serum 1,5-AG levels did not differ by gender significantly. When analyzed in the total study population, 1,5-AG correlates inversely with age significantly (p = 0.036). In subgroup analysis, in the control group, serum 1,5-AG level was also inversely correlated with age (p = 0.087). The best marker for the diagnosis of prediabetes and DM was fasting plasma glucose (FPG). 1,5-AG was not found to be effective for the diagnosis of DM. This study, contributes to our knowledge of the efficiency and cut-off values of 1,5-AG for the diagnosis of prediabetes and DM. In future, there is a need for larger studies with more standardized and commonly used measurement methods for 1,5-AG, in order to evaluate the efficiency of 1,5-AG for the diagnosis of prediabetes and DM.

## Background

Diabetes mellitus (DM) is a syndrome which insulin function or secretion or both impaired (Arrellano-Valdez et al. 2014). Prediabetes is the state between the normal blood glucose level and DM. The mechanism of prediabetes is insulin resistance and pancreatic beta cell disfunction (Pour and Dagogo-Jack 2011). Prediabetes is a risk for DM and cardiovascular disease (Twigg et al. 2007). Prediabetes has two basic types. One of them is IFG (impaired fasting glucose) and the other is IGT (glucose intolerance). In present fasting glucose, randomly plasma glucose with DM symptoms and 75 g OGTT is widely used for the diagnosis of DM. In 2009, ADA (American Diabetes Association) stated that HbA1c (glycated hemoglobin) could be used for DM diagnosis (International Expert Committee 2009). Glycated hemoglobin shows the average plasma glucose levels for the past 2–3 months. It is an advantage that fasting is not required for measurement of the glycated hemoglobin (McCance et al. 1994). Some authors suggests the use of the glycated hemoglobin in DM diagnosis (Kim et al. 2011; Saudek and Brick 2009), whereas the other do not agree this idea because of the low sensitivity levels of glycated hemoglobin (Selvin et al. 2009; Malkani and Mordes 2011). Fructosamine is created by the non-enzymatic glicolization of serum proteins such as albumine. Fructosamine shows the average plasma glucose of past 1–3 weeks. Glycated hemoglobin is more widely used for to measure plasma glucose levels; however fructosamine is superior to glycated hemoglobin in some conditions like hemoglobinopathy which glycated hemoglobin could be mistaken (Armbruster 1987; Koch 1996). HOMA-IR is actually used for to detect the insulin resistance (Yang et al. 2013). In some conditions that glycated hemoglobin can be wrong, that other indicators such as 1,5-anhydroglucitol (1,5-AG) is suggested to be used. 1,5-AG is a monosaccaride that exists in food. It is filtrated through kidneys and absorbed by renal tubules. The glucose afinity of proximale tubules is higher than the afinity to 1,5-AG. Because of this reason in the hyperglisemic state when glycosuria occurs, the reabsorption of 1,5-AG decreases and it is excreted by urine. So, the levels of plasma 1,5-AG decreases when the blood glucose level increases (Homa and Majkowska 2010; Stickle and Turk 1997). 1,5-AG shows the glucose levels of past 10–14 days (Stettle et al. 2008). In some studies 1,5-AG suggested as an indicator of blood glucose levels (Umeda et al. 1991). There are also some studies that suggest 1,5-AG can be used as a tool for DM diagnosis (Shah et al. 2009; Frattali and Wolf 1994)

## Aim

The aim of this study is to document the importance of 1,5-AG in the diagnosis of prediabetes and DM and compare 1,5-AG with glycated hemoglobin, fructosamine, fasting glucose and HOMA-IR in order to understand which one is the better diagnostic tool.

## Methods

The study is carried out in Ufuk University Dr.Rıdvan Ege Hospital in the city of Ankara, Turkey. Between April 2012 and December 2012, 128 patients who were performed 75 g OGTT, involved in study. The patients were joined the study from policlinics of internal medicine and endocrinology. The criteria to join the study were being 25 year old or older and having indication for OGTT. The exclusion criteria were, pregnancy, kidney disease (creatine levels 3 mg/dl or higher), liver disease, malabsorption syndromes, usage of steroids, polygala, tenuifolia, senega, alpha-glucosidase inhibitors, anemia, DM diagnosis before, usage of antidiabetic drugs and history of gastrectomy. This study is a cross-sectional analysis of an observational study. Ufuk University ethical board approval has been received. All participants gave informed consent. All of the ethical considerations have been strictly followed in accordance with the Helsinki declaration. All of the measurements were performed within a few days after the indication for OGTT. Participants who agreed to attend study gave blood samples between hours 08.00–09.00 after minimum 10 h of fasting. 75 g OGTT was performed and the patients were divided into 5 groups according to OGTT World Health Organization (WHO) criteria which were IFG, IGT, IFG+IGT, DM and control groups. From fasting glucose samples Glycated hemoglobin, fructosamine, total cholesterol, LDL, HDL, trigliseride, BUN, creatinine, CBC, AST, ALT, albumine, total protein, sedimentation, fasting insulin, fasting c-peptide levels were detected. Blood samples from each patient taken for 1,5-AG levels had stored at −83 cantigrat degree for the analysis day with ELISA method. Each patients arterial blood pressure were measured and demographic data were recorded. Waist circumference measurement executed from half of distance between inferior costa and spina iliaca anterior superior. Body mass index and HOMA-IR (fasting plasma glucose (FPG) (mg/dl) × fasting plasma insulin (μ/ml)/405) were calculated for each participant. The pancreas B cell function were calculated with the formula of 360 × fasting plasma insulin (μ/ml)/FPG (mg/dl) −63 %. The analysis of blood samples were performed at Ufuk University Dr. Rıdvan Ege Hospital Biochemistry Department Laboratuary. Glycated hemoglobin was measured with “Agilent 1100 Series” with “High Pressure Likid Chromatography” method whereas fructosamine was measured with “Cobas Integra 400 Plus” with spectrophotometric method. BUN, creatinine, HDL, LDL, trigliseride, total cholesterol, albumine, total proteine blood samples were detected with “Cobas Integra 800” with spectrophotometric method. The analysis of plasma samples for glucose levels were executed with hexocinase method by “Cobas Integra 800”. 1,5-AG is measured with “Cusabio Human 1,5-anhydroglucitol ELISA Kit” by “DSX Automated Elisa System” machine.

### Statistical analyses

Statistical analyzis executed with “IBM SPSS Statistics 20” programme. Firstly the distribution type of data analyzed. Data which showed normal distribution patern analyzed with ANOVA or *T* Test methods and results were presented as mean ± standard deviation. The dual comparison of the data which showed normal distrubution, TUKEY method was used. Data which did not show normal pattern were analyzed with Cruscal-Wallis or Mann–Whitney U methode and results were presented as mean and 95 % confidence interval. Dual comparison of data which do not showed normal pattern executed with “Bonferroni corrected Mann–Whitney U” methode. Spearman corelation analyzes performed to investigate serum 1,5-AG and other variables. In the comparison of the power of the diagnostic tools, ROC (Receiver Operating Characteristics) curve is used. Threshold level of the diagnostic tools determined as the point in the ROC curve which is closest to sensitivity value 100 % and false positive value 0 %. HOMA-IR calculated as “FPG (mg/dl) × fasting insulin (μU/ml)/405” and HOMA-B calculated as “360 x fasting insulin (μU/ml)/(FPG (mg/dl)-63)”. Statistical significance point set to p < 0.05.

## Results

**Fig. 1:**
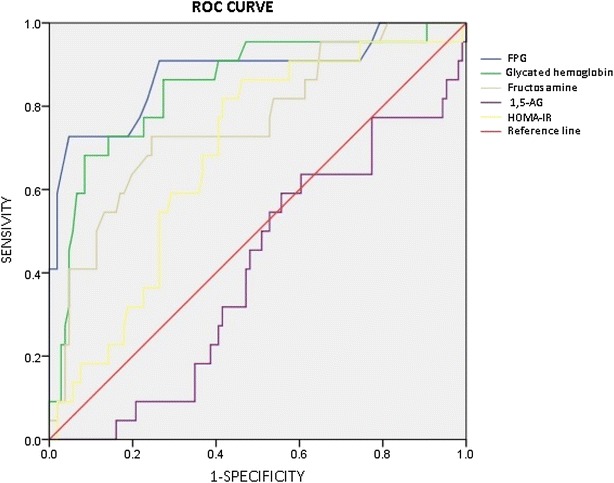
Comparison of markers for the diagnosis of DM

Between April 2012 and December 2012, 128 participants who applied to endocrinology or internal medicine clinics of Ufuk University Dr. Rıdvan Ege Hospital, enrolled in this study. All participants had indication for 75 g OGTT and gave informed consent. Mean age of participants were 53.0 ± 12.2. 48 participant (37.5 %) were male, 80 participant (62.5 %) were female. IFG, IGT, IFG+IGT groups together evaluated as “prediabetic group”. Control group has the most participant with 38 people. Mean age of participants was highest in diabetic group. Distribution of participant according to gender was not significantly different between groups. Data of participants were described in Table 1. We investigated the 1,5-AG levels of participants in control, prediabetes, DM groups. 1,5-AG levels of prediabetic group was higher than diabetic group (p = 0.039). Also, 1,5-AG levels were higher in control group than prediabetic group (p = 0.064). We examined fructosamine and glycated hemoglobin levels in control, prediabetic and diabetic groups. Mean plasma glycated hemoglobin levels were higher in prediabetes group than control group (p < 0.001). Also, plasma glycated hemoglobin levels in DM group were higher than prediabetes group (p < 0.001). Mean serum fructosamine levels were higher in prediabetes group than control group (p = 0.013). Also, mean serum fructosamine levels were higher in DM group than prediabetes group (p = 0.003). We investigated the difference of plasma 1,5-AG levels according to gender. There is no significant difference of blood 1,5-AG levels according to gender (p = 0.813). We investigated the 1,5-AG levels according to age. In the total study population 1,5-AG levels found inversly related to age (p = 0.036). In subgroup analysis, the most significant inverse correlation of 1,5-AG and age found in control group (p = 0.087). When genders were separetely analyzed, in female cases the inverse correlation between age and 1,5-AG was significant (p = 0.035), however in male cases this correlation was not significant (p = 0.637). We examined the relationship between 1,5-AG and 2 h plasma glucose levels however we did not find any significant relationship between 1,5-AG and 2 h plasma glucose. Then, we evaluated and compared the FPG, glycated hemoglobin, fructosamine, 1,5-AG and HOMA-IR in diagnosis of prediabetes. Participants who underwent OGTT evaluated according to WHO criteria. 68 cases labelled as prediabetes. Table 2 shows the comparison of FPG, HbAac, fructosamine, 1,5-AG and HOMA-IR as a diagnostic tool in prediabetes. FPG has the largest area under curve. Glycated hemoglobin, fructosamine, HOMA-IR and 1,5-AG follows FPG respectively. Fructosamine and HOMA-IR had the same area under curve. Optimal threshold value for diagnostic tools in prediabetes diagnosis provides 72.1 % sensitivity, 86.6 % specificity for FPG. The other values listed in Tables 2 and 3. We evaluated and compared the FPG, glycated hemoglobin, Fructosamine, 1,5-AG and HOMA-IR for the diagnosis of DM. Participants who underwent OGTT evaluated according to WHO criteria. 22 cases labelled as DM. Table below shows the comparison of FPG, HbAac, fructosamine, 1,5-AG and HOMA-IR as a diagnostic tool in DM. FPG has the largest area under curve. Glycated hemoglobin, fructosamine, HOMA-IR and 1,5-AG follows FPG respectively. Area under curve of 1,5-AG remained below of the reference line (Fig. 1). The other values listed in Tables 2 and 3. We evaluated and compared the FPG, glycated hemoglobin, fructosamine, 1,5-AG and HOMA-IR in diagnosis of glucose intolerance (DM+prediabetes). Participants who underwent OGTT evaluated according to WHO criteria. 22 cases labelled as DM and 68 cases labelled as DM. Table 2 shows the comparison of FPG, HbAac, fructosamine, 1,5-AG and HOMA-IR as a diagnostic tool in DM and prediabetes. FPG has the largest area under curve. Glycated hemoglobin, fructosamine, HOMA-IR and 1,5-AG follows FPG respectively. The other values listed in Tables 2 and 3.

**Table 1:** Data of participants

Groups	(1) CONTROL (n = 38)	(2) IFG (n = 24)	(3) IGT (n = 23)	(4) IFG + IGT (n = 21)	(5) DM (n = 22)	p value
Age	*(4,5)*50.1 ± 9.0	*(4,5)*48.5 ± 13.2	*(5)*51.6 ± 11.5	*(1,2)*57.4 ± 11.5	*(1,2,3)*60.3 ± 13.4	0.002
Gender (M/F)	14/24	7/17	10/13	8/13	9/13	0.881
Fasting PG (mg/dl)	*(2,4,5)*89.2 (87.0–91.4)	*(1,3,5)*106.0 (103.7,108.4)	*(2,4,5)*91.9 (89.8–94.0)	*(1,3,5)*107.0 (104.7–109.4)	*(1,2,3,4)*128.2 (108.1–148.3)	<0.001
30.min PG (mg/dl)	*(2,4,5)*150.2 ± 27.4	*(1,5)*175.1 ± 39.9	*(4,5)*153.7 ± 28.7	*(1,3,5)*183.3 ± 23.2	*(1,2,3,4)*215.9 ± 60.6	<0.001
60.min PG (mg/dl)	*(2,3,4,5)*142.9 ± 41.0	*(1,4,5)*182.4 ± 49.5	*(1,5)*185.7 ± 36.1	*(1,2,5)*209.9 ± 29.4	*(1,2,3,4)*275.3 ± 68.0	<0.001
90.min PG (mg/dl)	*(3,4,5)*117.3 ± 30.2	*(3,4,5)*140.3 ± 33.5	*(1,2,5)*173.3 ± 36.5	*(1,2,5)*195.9 ± 30.4	*(1,2,3,4)*282.3 ± 87.3	<0.001
120.min PG (mg/dl)	*(3,4,5)*100.6 (93.5–107.8)	*(3,4,5)*113.9 (105.5–122.3)	*(1,2,5)*160.7 (153.3–168.2)	*(1,2,5)*169.3 (162.2–176.4)	*(1,2,3,4)*261.7 (220.0–303.5)	<0.001
HbA1c (%)	*(2,4,5)*5.29 ± 0.48	*(1,4,5)*5.79 ± 0.41	*(4,5)*5.63 ± 0.57	*(1,2,3,5)*6.22 ± 0.86	*(1,2,3,4)*6.72 ± 0.99	<0.001
Fructosamine (µmol/l)	*(5)*227.2 (221.2–233.3)	238.0 (228.0–248.0)	*(5)*229.6 (219.3–239.9)	248.1 (233.4–262.8)	*(1,3)*269.1 (240.9–297.4)	<0.001
1,5-Anhydroglucitol (nmol/ml)	73.2 (27.5–118.9)	59.8 (39.4–80.1)	54.4 (32.1–76.7)	51.9 (34.6–69.3)	33.3 (22.2-44.5)	0.189
Total cholesterol (mg/dl)	196.0 ± 37.6	193.2 ± 36.4	199.2 ± 32.0	204.2 ± 29.6	192.0 ± 35.2	0.783
LDL (mg/dl)	123.0 ± 29.8	119.6 ± 32.9	126.7 ± 22.7	128.2 ± 25.4	116.9 ± 31.2	0.664
HDL(mg/dl)	47.1 ± 13.7	50.2 ± 13.5	42.1 ± 13.7	52.1 ± 18.2	44.4 ± 11.6	0.124
Triglyceride (mg/dl)	134.2 (113.3–155.2)	125.3 (100.5–150.1)	175.3 (130.1–220.5)	139.7 (101.2–178.2)	168.7 (125.7–211.8)	0.380
Fasting insulin (µu/ml)	15.00 (9.73–20.27)	16.27 (13.19–19.36)	11.54 (8.92–14.16)	15.51 (12.70–18.33)	14.31 (11.58–17.04)	0.030
Fasting c-peptide (ng/ml)	3.44 (2.92–3.97)	3.80 (3.10–4.51)	*(5)*2.98 (2.54–3.42)	3.44 (3.08–3.81)	*(3)*3.76 (3.43–4.09)	0.021
HOMA-IR	*(2,4,5)*3.37 (2.10–4.63)	*(1,3)*4.28 (3.43–5.13)	*(2,4,5)*2.63 (2.02–3.25)	*(1,3)*4.10 (3.35–4.86)	*(1,3)*4.55 (3.47–5.63)	<0.001
HOMA β	*(5)*208.1 (149.1–267.2)	136.7 (111.4–162.0)	144.1 (113.4–174.7)	127.9 (104.3–151.6)	*(1)*93.1 (73.3–112.9)	<0.001
Systolic BP (mmHg)	122.1 (117.4–126.7)	127.0 (115.6–138.5)	124.7 (116.3–133.2)	129.5 (120.1–138.9)	124.0 (117.1–131.0)	0.809
Diastolic BP (mmHg)	78.2 (75.1–81.4)	79.5 (74.0–85.0)	78.6 (73.2–84.1)	80.1 (74.2–86.0)	74.5 (69.8–79.2)	0.598
Mean BP (mmHg)	92.8 ± 10.7	95.4 ± 17.0	94.0 ± 14.4	96.6 ± 14.5	91.0 ± 11.0	0.670
Height (cm)	162.9 ± 7.8	164.8 ± 8.1	164.6 ± 9.5	162.6 ± 7.9	162.0 ± 9.7	0.748
Weight (kg)	74.8 ± 14.7	84.2 ± 17.6	72.4 ± 13.8	78.5 ± 17.0	79.0 ± 16.7	0.099
Body mass index	*(2,4,5)*26.2 ± 5.2	*(1,3)*30.9 ± 5.7	*(2,5)*26.6 ± 4.4	*(1)*29.6 ± 6.1	*(1,3)*30.2 ± 7.1	0.004
Waist circumference (cm)	91.0 ± 12.2	97.9 ± 10.6	88.3 ± 10.4	92.8 ± 13.6	94.0 ± 11.9	0.069

Data which do not show normal pattern analyzed with Cruscal-Wallis or Mann–Whitney U methode and results were shown mean and 95 % confidence interval. Dual comparison of data which do not showed normal pattern executed with “Bonferroni corrected Mann–Whitney U” methode. Near data (×) plot put to indicate which group was significantly different against other group. (1. Groups named with numbers as: Control group 1, IFG group 2, IGT group 3, IFG+IGT group 4, and DM group 5). (2. Fasting insulin value was not significant in any comparison)

* Data which showed normal distribution patern analyzed with ANOVA or T Test methode and results showed as mean ± standard deviation. The dual comparison of the data which showed normal distrubution, TUKEY methode is used

**Table 2:** Evaluation and comparison of FPG, glycated hemoglobin, fructosamine, 1,5-AG, HOMA-IR in prediabetes, DM and glucose intolerance diagnosis

Diagnostic tools	Area under curve		
	Prediabetes^a^	DM	Glucose intolerance
FPG	0.867	0.880	0.889
Glycated hemoglobin	0.775	0.848	0.815
Fructosamine	0.646	0.761	0.696
1,5-AG	0.609	0.405	0.582
HOMA-IR	0.646	0.683	0.683

Total study population (128 patients) was analyzed for the evaluation of the markers as a diagnostic tool for DM and glucose intolerance

^a^DM patients were excluded (106 patients were analyzed) for the evaluation of the markers as a diagnostic tool for Prediabetes

**Table 3:** The comparison of sensitivity and specificity of threshold values of diagnostic tools for prediabetes, DM and glucose intolerance diagnosis

	Diagnostic tool	Optimal threshold value^a^	Sensitivity	Specificity
Prediabetes^b^	FPG (mg/dl)	96.50	0.721	0.868
	Glycated hemoglobin (%)	5.60	0.706	0.816
	Fructosamine (µmol/l)	230.50	0.676	0.579
	1,5-AG (nmol/ml)	38.79	0.618	0.711
	HOMA-IR	3.00	0.618	0.684
DM	FPG (mg/dl)	112.50	0.727	0.953
	Glycated hemoglobin (%)	5.99	0.864	0.726
	Fructosamine (µmol/l)	249.50	0.727	0.755
	1,5-AG (nmol/ml)	30.97	0.591	0.443
	HOMA-IR	3.21	0.818	0.585
Glucose intolerance	FPG (mg/dl)	97.50	0.744	0.947
	Glycated hemoglobin (%)	5.60	0.767	0.816
	Fructosamine (µmol/l)	233.50	0.656	0.632
	1,5-AG (nmol/ml)	33.22	0.633	0.658
	HOMA-IR	3.00	0.678	0.684

Total study population (128 patients) was analyzed for the evaluation of the markers as a diagnostic tool for DM and glucose intolerance

^a^Threshold value for each diagnostic tool calculated as the nearest point of ROC curve that sensitivity 100 %, false positivity is 0 %

^b^DM patients were excluded (106 patients were analyzed) for the evaluation of the markers as a diagnostic tool for Prediabetes

## Discussion

Early diagnosis in prediabetes and DM can reduce the mortality and morbidity of the disease. The reference test for the diagnosis of prediabetes and DM is OGTT according to the WHO recommendations. For screeening of glucose intolerance, 2 h post-OGTT glucose level is used. Fasting condition of the patient is needed for the OGTT and FPG tests. For this reason application of these methods can be challenging for doctor and patient. Also if the patient is not in fasting condition, it is required to come to hospital for a second time, thus making the situation tiring and expensive. Therefore, alternative diagnostic tools which do not reguire fasting condition, may be preferred. Glycated hemoglobin, fructosamine, 1,5-AG are diagnostic tools which do not reguire fasting (Shah et al. 2009). Many studies have investigated the role of the 1,5-AG in DM. In some studies, the efficiency of 1,5-AG found higher than glycated hemoglobin whereas more studies suggests glycated hemoglobin is better because 1,5-AG has wide range of normal distrubution. As a result, the role of 1,5-AG in diagnosis of DM remained uncertain (Robertson et al. 1993; Shirasaya et al. 1999; Yamanouchi et al. 1991; Yamanouchi et al. 2001).

In many reports, it is found that there is a statistically significant inverse relationship between plasma 1,5-AG level and post OGTT second hour plasma glucose (Yamanouchi et al. 1988; Goto et al. 2011). However in our study we did not find any relationship between plasma 1,5-AG and post OGTT second hour glucose levels. The cause of this can be explained by the relatively low number of participants in our study in comparison to the other studies. There are some studies that investigates the role of 1,5-AG in the diagnosis of DM and prediabetes. Our study has similarities and differences from these studies. Firstly in our study, the mean plasma 1,5-AG levels were found higher in control group than prediabetic group. Also the 1,5-AG levels were found higher in prediabetic group than diabetic group (Shirasaya et al. 1999; Won et al. 2009). In many other studies it is suggested that the normal distribution range of 1,5-AG is so wide that it limits the usage of 1,5-AG in DM screening (Robertson et al. 1993; Yamanouchi et al. 1991; Won et al. 2009). Similarly, 1,5-AG has distributed in a wide range in our study, thus with the additive effect of limited case numbers we did not get statistical significance. The importance of the glisemic markers in diagnosis can be investigated many ways. Selectivity index or ROC curve method can be used (Koch 1996; Shirasaya et al. 1999; Yamanouchi et al. 1991; Goto et al. 2011). We preferred the ROC curve in our study. In a study conducted by Yamanouchi et al. selectivity index was used to investigate the most important marker in DM diagnosis. 1,5-AG was found as the best marker, glycated hemoglobin and fructosamine followed it respectively (Yamanouchi et al. 1991). In another study, the most important markers found as FPG, 1,5-AG, glycated hemoglobin, and fructosamine respectively (Shirasaya et al. 1999). In a different study the most important markers in DM screening found as glycated hemoglobin followed by 1,5-AG, FPG and HOMA-IR (Koch 1996). In our study the area under curve of 1,5-AG was lower than reference line so it is not suitable to use it as a marker of diagnosis of DM. In our study the most important marker was found as FPG in diagnosis for prediabetes, DM and glucose intolerance. The FPG is an important diagnostic tool in DM and it should be preferred as first test in screening of prediabetes and DM. In our study glycated hemoglobin was found as best marker for prediabetes and DM diagnosis, which does not require fasting state. Recently, glycated hemoglobin is suggested as a marker of DM which could be used alone because of the improvements in standardization of measurement techniques. Our study supports this issue, as we found Glycated hemoglobin as the second valuable marker in the DM diagnosis. In our study, 1,5-AG did not found as valuable a diagnostic marker in DM and it was found as the least valuable marker in prediabetes and glucose intolerance. The small number of samples compared to the wide distribution range can be the reason for that finding. In a study, the efficiency of 1,5-AG, glycated hemoglobin and fructosamine in prediabetes and DM compared with optimal threshold levels (Shirasaya et al. 1999). Optimal threshold levels were decided as the point on the ROC curve which is nearest to 100 % sensitivity and 0 % false positivity. According to this, when 104 nmol/ml was taken as threshold for 1,5-AG, 83.8 % sensitivity and 84.6 % specificity values were obtained for type 2 DM diagnosis. When 5.6 % was taken as threshold for glycated hemoglobin, 83.8 % sensitivity and 79.4 % specificity values were obtained for type 2 DM diagnosis. When 256 µmol/l was taken as threshold for fructosamine, 70.3 % sensitivity and 79.9 % specificity values were obtained for type 2 DM diagnosis (Shirasaya et al. 1999). In the same study the thresholds were calculated for type 2 DM+IGT. When 134 nmol/ml was taken as threshold for 1,5-AG, 62.7 % sensitivity and 61.0 % specificity values were obtained. When 5.4 % was taken as threshold for glycated hemoglobin, 53.2 % sensitivity and 68.6 % specificity values were obtained. When 244 µmol/l was taken as threshold for fructosamine, 61.2 % sensitivity and 55.4 % specificity values were obtained (Shirasaya et al. 1999). In another study, thresholds of 1,5-AG, glycated hemoglobin, fructosamine were calculated by selectivity index (Yamanouchi et al. 1991). When 85.2 nmol/ml was taken as threshold for 1,5-AG, 84.2 % sensitivity and 93.1 % specificity values were obtained for DM diagnosis. When 6.2 % was taken as threshold for glycated hemoglobin, 67.5 % sensitivity and 92.7 % specificity values were obtained for DM diagnosis (Yamanouchi et al. 1991). In a different study, in type 2 DM screening of insulin resistant subgroup, threshold level for 1,5-AG was taken as 103.56 nmol/ml which gives 96 % sensitivity and 88 % specificity values (Koch 1996). In the total study population, the threshold value of the glycated hemoglobin was taken as 5.7 % which gives 86 % sensitivity and 85 % specificity. In the total study population, the threshold value of HOMA-IR was taken as 7.9 which give 62 % sensitivity and 70 % specificity. Again in the total study population, the threshold value of FPG was taken as 104 mg/dl which gives 88 % sensitivity and 93 % specificity (Koch 1996). In our study we have investigated markers for prediabetes, DM and glucose intolerance diagnosis. After the results, we understand 1,5-AG is not a suitable marker for the diagnosis of DM. Also, the effectiveness of 1,5-AG was the lowest in the diagnosis of prediabetes and glucose intolerance. These findings opposed the past studies in literature. There can be several reasons for this. Firstly, other studies demonstrated in much larger patient cohorts. Secondly, the plasma 1,5-AG has a wide normal distribution range and our patient cohort was relatively small for this wide distribution range. Thirdly, 1,5-AG measurement technic and equipments differs between studies. Lastly, all study participants had an indication for OGTT testing which means that the control group was also selected in the basis of diabetes risk, thus explaining the weaker discriminatory results as compared to other studies. In our study all measurements were performed a few days after the indication for OGTT. Therefore we have prevented our study data to be influenced from possible lifestyle changes by participants after hearing the indication for “diabetes testing”. In the literature there are different threshold levels of 1,5-AG for DM diagnosis. These values differ between studies and there is no consensus on these values. Values in our study differ from other studies too. There could be several reasons for this. Firstly, there can be all reasons that are mentioned above (size of patient cohorts, wide distribution range, etc.). Secondly, plasma 1,5-AG levels could differ due to eating habits and race (Herman et al. 2009; Koga et al. 2010). However there is no study about 1,5-AG for DM diagnosis in our country which we could refer. As a result, there is no consensus on 1,5-AG threshold levels for DM and prediabetes, and our study contributes literature by offering threshold values for 1,5-AG in the diagnosis of DM, prediabetes and glucose intolerance.

To conclude, although there are several studies in the literature about the relationship between 1,5-AG and DM diagnosis; our study is the first study about this issue that is demonstrated in our country. Also, this is first study about in our country that is performed clinically and prospectively. In respect to this, our study may represent the threshold values of 1,5-AG in our country that can be referred by other studies that will be conducted future. The limitations of our study was the relatively small patient cohort compared to wide distribution range of 1,5-AG.
